# Ethorobotics: A New Approach to Human-Robot Relationship

**DOI:** 10.3389/fpsyg.2017.00958

**Published:** 2017-06-09

**Authors:** Ádám Miklósi, Péter Korondi, Vicente Matellán, Márta Gácsi

**Affiliations:** ^1^Department of Ethology, Eötvös Loránd UniversityBudapest, Hungary; ^2^Magyar Tudományos Akadémia – Eötvös Loránd University Comparative Ethology Research GroupBudapest, Hungary; ^3^Department of Mechatronics, Optics and Information Engineering, Budapest University of Technology and EconomicsBudapest, Hungary; ^4^Departamento Ingeniería Mecánica, Informática y Aeroespacial, Universidad de LeónLeón, Spain

**Keywords:** social robotics, ethology, human-robot interaction, niche, social competence, dog, uncanny valley

## Abstract

Here we aim to lay the theoretical foundations of human-robot relationship drawing upon insights from disciplines that govern relevant human behaviors: ecology and ethology. We show how the paradox of the so called “uncanny valley hypothesis” can be solved by applying the “niche” concept to social robots, and relying on the natural behavior of humans. Instead of striving to build human-like social robots, engineers should construct robots that are able to maximize their performance in their niche (being optimal for some specific functions), and if they are endowed with appropriate form of social competence then humans will eventually interact with them independent of their embodiment. This new discipline, which we call *ethorobotics*, could change social robotics, giving a boost to new technical approaches and applications.

## The More Human-Like the Better?

Motto: “You climb to reach the summit, but once there, discover that all roads lead down.”

Stanislaw Lem, The Cyberiad

Social robotics is the science for developing and building robots that can be integrated into human groups, and are able to engage in complex social interactions with humans, including communication and collaboration (e.g., Fong et al., 2003; Dautenhahn, 2007).

The recent increased interest by the media to introduce and popularize such robots to the public (e.g., Saya) and general interest in science fiction (e.g., AI, Robocop) seems to make both lay persons and many scientists to believe that social robotics should produce robots (so called androids) that match perfectly humans both in their embodiment (e.g., DiSalvo et al., 2002) and in their communicative and problem solving skills (some improved version of C-3PO). Although the emergence of everyday social robots on the markets is still decades away, marketing pressure, grant agencies (in the United States, EU, and China), and the challenges of engineering also push applications toward building human-like robots.

Subjectively one may feel that humans like to be and interact with agents of closely similar kind and may avoid more machine-like creatures. However, the only serious hypothesis, which was put forward by Mori (1970), argues the opposite: the more similar robots are to humans the more humans avoid them.

## The Rethinking of the ‘Uncanny Valley’ Hypothesis and its Predictions

**FIGURE 1:**
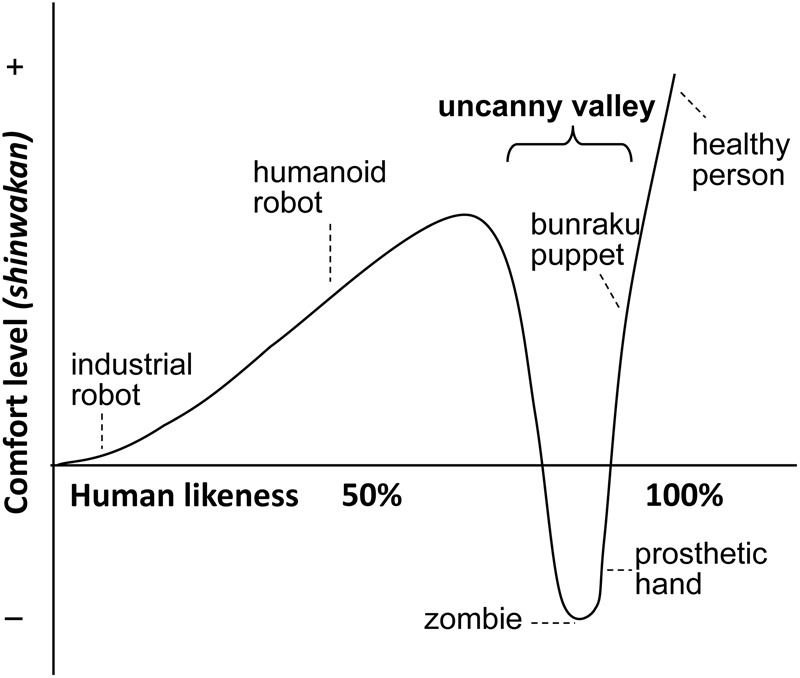
A modified reproduction of Mori’s (1970) original ‘uncanny valley’ hypothesis by showing only the reaction to moving agents.

The ‘uncanny valley’ hypothesis articulated by Mori in 1970 was the first theoretical evaluation of the predicted relationship between humans and non-living agents, including robots. **Figure 1** presents a modified reproduction of Mori’s (1970) original idea by showing the humans’ reaction only to moving agents. It is assumed that social robots getting very similar to humans (measured by some complex variable) are being more and more rejected by people. Very similar robots are rejected much more than less similar ones. Social robots may never reach the ‘Maximum peak’ which represents humanness. Implicitly this figure also suggests that social robotics develops from left to right aiming specifically at designing human-like robots. Thus the X axis represents both “human likeness” and “time.”

Mori’s hypothesis suggests a complex relationship between the agent’s (biological or artificial) similarity to a human and the human’s affinity toward the agent. Accordingly, the dependent variable (in Japanese ‘shinwakan’), called affinity (MacDorman and Minato, 2005) has two local maximum values. The first one on the left (**Figure 1**) is referred to as the “Medium Peak.” It emerges at a point where similarity between the agent and a typical human is substantial but still relatively low (approx. 60–75%). The other one is at the right part of the figure when the agents reach (nearly) perfect similarity with humans. This is the “Maximum Peak.” Most importantly, it is claimed that for a narrow range of very close similarity to humans, values of affinity will obtain very low or even negative values, labeled as the uncanny valley.

In the original paper Mori left open the question of causation, and subsequent scientific discussions focused on either (1) evolutionary explanations (e.g., avoidance of threat, or death; see MacDorman and Ishiguro, 2006; Moosa and Minhaz Ud-Dean, 2010), (2) developmental effects (e.g., babies show this effect only after 12 months of age; Lewkowicz and Ghazanfar, 2012), or (3) perceptual and mental mechanisms (e.g., activation of competing mental representations; Chen et al., 2009; Ferrey et al., 2015). While these explanations are not mutually exclusive they all assume that the phenomenon is specific to humans (or non-human primates) (MacDorman et al., 2009; Steckenfinger and Ghazanfar, 2009) and researchers investigate it only in relation to artificial creatures (cf. robots) (Mathur and Reichling, 2016).

One may consider that the phenomenon may have a more wide-spread biological (functional) basis, the recognition of which leads to a different perspective. Here we argue that the present trend in social robotics is misguided. We show that an ethological approach, considering functional aspects of behavior and human-robot interaction, can provide a more plausible theoretical background for social robotics. We aim to establish an interdisciplinary science of ethorobotics, which relies on evolutionary, ecological, and ethological concepts for developing social robots. We suggest that while the similarity of the agent’s characteristics may enhance the efficiency of the interactions, the social identification/categorization of the agent also plays a crucial role in respect of affinity and expectations.

## The Importance of Recognition of Others

We propose that in humans the avoidance of very closely similar others reflects a more widely distributed skill in animal species, which is aimed to precisely categorize and recognize other potentially significant biological agents. The specific function of this ability depends on the ecology of the species but this process is invaluable for survival (Mateo, 2004). In general biological agents should be able to discriminate others at three different levels: (1) conspecifics (same species) versus heterospecifics (other species, e.g., predators); (2) familiar conspecifics (e.g., group members) versus unfamiliar conspecifics (e.g., strangers/intruders); (3) familiar conspecifics versus individuals (e.g., mate, friends, and pups). The rapid and precise discrimination of others is important because it determines what kind of actions should be taken and what kind of responses could be expected. Animals may rely on different set of features (e.g., visual, auditory and olfactory) for this discrimination but generally it can be assumed that the computational need is the highest at the 3^rd^ level.

Biological agents achieve this performance by being sensitive to some simple but specific pattern of cues (e.g., sign stimuli) early in their development, and this attraction provides the basis for further learning about the peculiarities of others. Such learning usually takes place during a specific sensitive phase when some neural structures acquire selective responsiveness to recognize and discriminate specific set of cues. Such perceptual learning is based on selective elimination of not-stimulated pre and post-synaptic connections. Although such learning can take place also later in development or adulthood, the stronger and less reversible effects probably happen when the neural system matures. The ability to discriminate others has been investigated in several species (Colgan, 1983), and also on humans.

### Sensitive Period of Social Recognition in Humans

Recently, it has been hypothesized that early experience with human faces provides the basis of the uncanny valley effect in infants (Ferrey et al., 2015). The comparison of 6 to 12 month old infants showed that only the oldest group avoided unrealistic faces.

It has been long known that few hour old newborns show preference toward face-like patterns (Johnson et al., 1991). More recent results have indicated that 3-day-old newborns look longer at faces gazing at them directly, and they also prefer to look at faces presenting two eye-like patterns on the top rather than on the bottom (Farroni et al., 2005). It seems that there is a genetically canalized preference for some visual features (sign stimuli) that make the infant focus on the (human) face. This interest helps the infant to learn about other components of the face that is made possible by the parallel improvement of visual and neural processing (e.g., Gliga and Csibra, 2007; Pascalis and Kelly, 2009). As a result infants become experts in discriminating and recognizing individuals from the same category (familiar faces in the group). Babies are much better in making such discriminations in the case of their own race than in other races (‘other-race’ effect; e.g., Kelly et al., 2009), although this effect is smaller if babies are exposed to members of different races early on (Sangrigoli and De Schonen, 2004).

This natural process of emerging social recognition in humans suggests that only by massively exposing babies to (future) social robots can we avoid that they ‘fall in the uncanny valley.’ Such forced exposure seems unrealistic and would be also unethical, moreover, it could also confuse the social recognition system of humans, and lead to misguided social and sexual preferences.

## Re-Interpretation of the ‘Uncanny Valley’

We argue that in Mori’s landscape, the similarity measure (*X*-axis) relates to the interaction of heterogenic agents when one type of agent is used as point of reference. This is equivalent to a biological scenario with conspecifics and heterospecifics. Thus the Medium Peak refers to interactions with a specific group of heterospecifics that share many attributes with humans (e.g., domesticated animals) and the Maximum Peak refers to interaction among conspecifics (**Figure 1**). Note that heterospecific agents represent a much larger and diverse category than conspecific agents, and many heterospecific agents fall to the left from the Medium Peak. For example, from the humans’ point of view dogs and Rhesus monkeys can be both placed on an arbitrary *similarity* scale on Mori’s figure but it is questionable whether the same measure could be applied to *familiarity* with humans.

Importantly, the mental and behavioral mechanisms activated in the case of the Medium Peak and Maximum Peak are quite different, because biological agents possess a dedicated mechanism to detect individuals belonging to their own species but probably much less detailed discrimination is needed in the case of very different heterospecific species. Thus in the case of the Maximum Peak (distinguishing among conspecifics) the agent has to be more choosy and focused than when contacting heterospecific agents (Medium Peak). Biologically speaking this means that members of a species must avoid to get in close contact with non-conspecifics, e.g., hybrids, or closely related species because such mistakes can be fatal, especially with regard to reproduction (mating with hybrids reduces the fitness). This interpretation fits well with the depiction of the figure in which the Maximum Peak has a much narrower basis then the Medium Peak. Intuitively this suggests that conspecifics are evaluated more selectively then heterospecifics.

### Strategies for Social Robotics

In the light of recent research on social recognition learning (e.g., Lewkowicz and Ghazanfar, 2009), Mori’s hypothesis offers two options for developing optimal social robots. Social robots should achieve perfect humanness or humans (infants) should be exposed to social robots as soon as possible (before 1st year of age), which would probably decrease later uncanny feelings toward them. While the first option is quite unrealistic and counterintuitive (see also below), the second option may lead to serious problems because the exposure to such social robots during the sensitive period of infant development could lead to misguided learning about the human species, confusing species recognition and preferences at some later life (see debate initiated by Sharkey and Sharkey, 2010). Humans socialized as infants with robots (e.g., Tanaka et al., 2007) may prefer them later as social companions or sexual partners (Levy, 2010).

### Androids and *Trans*-Humans

Let’s assume for a moment that modern information technology continues to develop at least with the speed we have experienced in the last two decades. Then, there is little doubt that this technology will be able to surpass biologically evolved human traits in social robots, partly including new features not present in humans or any other naturally evolved agent. Just one example: gaze following is an automatic skill by which a bystander can perceive the focus of interest of the subject. Thus the head turn of one subject elicit head turn in others. A wide range of mammals and birds share this skill, which is based on visual perception and rapid processing of head orientation and movement. While such ability can be easily mimicked in an android robot, there is technically no restriction (even today) to equip a social robot with 360° vision capacities (just like in jumping spiders). This skill is certainly more advantageous for the robot but very likely it will change also how the robot behaves (no need to turn to follow the other’s gaze) and also how it processes visual information. Thus it is not difficult to envisage that even very much human-like robots may at some point over-perform and transcend human performance.

**FIGURE 2:**
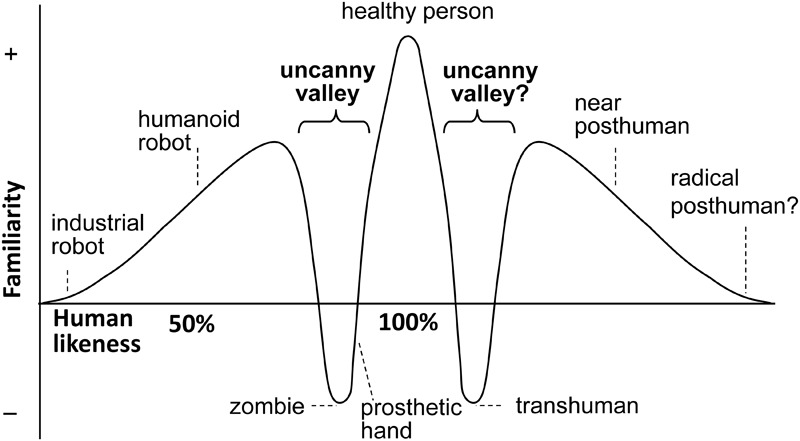
An extended version of Mori’s idea by Jamais Cascio (from http://www.openthefuture.com/2007/10/the_second_uncanny_valley.html). The second valley shows a similar effect related to robots evolved *from* perfect humanlike agents, as they become *less* similar to humans – following the path of *trans-*human and, eventually, *post-*human robots. The hill after the valley is when differentiation is strong enough to create a new category.

Thus “perfect” human-like robots would represent only a relatively short and transient period in the technical development of social robots, which would be followed by robots to which some people may refer to as “*trans*-humans” during a transitional period and then moving away from human likeness, as “post-humans” (see Jamais Cascio unpublished source^1^). **Figure 2** shows this extended version of the original idea, indicating that technical development may not end at reaching maximum humanness and social robots may “fall” into a second uncanny valley. For today’s social robotics this situation presents a real paradox.

In this sense, post-humans can be envisioned as “improved” humans but some of these agents may also fall into another uncanny valley to the right side of the “healthy person.” For example, it has been shown that humans may have problems in predicting the behavior of robots that look like us but behave differently (Saygin et al., 2012).

Thus, Mori’s hypothesis can be extended to a symmetrical landscape where there are two uncanny valleys on both sides of “perfect humanness” and humans may avoid both the lesser and the overly humanlike robots. Looking at this landscape it becomes clear that after the Maximum Peak has been reached there would be a narrow range of biological and artificial humans, in a largely extended world of heterospecific agents. Thus the notion of convergence in the direction of perfect humanness should be replaced by a more general view of divergence with regard to artificial systems, notwithstanding that such divergent processes may parallel a development of a specific class of agents which show very close resemblance to humans, and some of which may be able to evade the biological and cultural mechanisms of human social recognition system.

In summary, the paradox of the uncanny valley is that passing the valley successfully does not seem to solve the problem of social robotics because it is likely that robots will soon fall into another uncanny valley and/or in any case they will diverge from humanness. In addition, such *trans*-human robots that achieve or transcend human performance would very likely disrupt typical (natural) human social systems (Kubinyi et al., 2010).

## Ethological Approach to Social Robotics

The ethological approach is centered on the function of behavior in relation to the specific environment in which the species evolved (Tinbergen, 1963). The application of this general concept to social robotics means that the robot should have a function, and in terms of embodiment, behavior, and problem solving (cognitive) abilities it should fit its specific environment. Instead of aiming to build more and more human-like robots and trying to “climb” the Maximum Peak, we may start robot construction by determining their function and their environment and design the must suited agent independently from its similarity to humans. Note that robot engineering can proceed by ‘jumps’ from one type of agent to a radically different one because it is not constrained by evolutionary continuity like biological agents. Moreover, humans may be not adequately ‘designed’ for a range of tasks thus uncritical copying of humans could turn out as wasted effort.

### Solving the Paradox of the ‘Uncanny Valley’ Hypothesis

**FIGURE 3:**
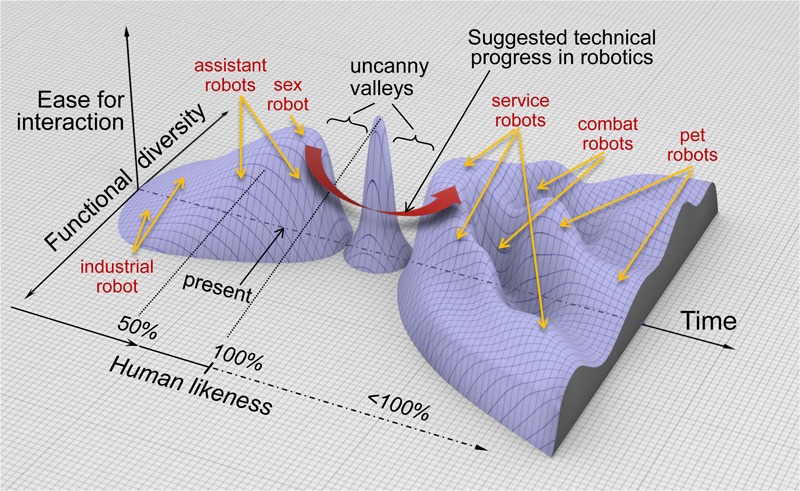
An ethorobotic concept of emerging human-robot interaction. Based on Mori’s idea, the present situation and the envisaged progress of social robotics are shown in a three-dimensional space to separate human-likeness, functionality and ease of interaction. After the peak and the second uncanny valley, robots are likely to evolve into a diversity of morphologies and behaviors that, depending on their functions, gradually move away from perfect human likeness. The wide curved arrow indicates the possible detour for social robotics by moving directly from the present state to less humanlike robots with diverse functionality retaining high-level capacity for social interaction with humans. The labels on the terrain are only for informative purposes and do not necessarily refer to actual existing robots.

With regard to the uncanny valley metaphor this would mean that we go around the Maximum Peak and avoid the uncanny valley on the other side (**Figure 3**). Ethologically, such a robot is occupying a different niche that is created by its specific function. This approach has several beneficial consequences: (1) robots can have their own evolution without interfering specifically with that of humans; (2) robots survive only if their niche exists and die out if they have not performed well to the expectation of humans; (3) no competition emerges between humans and robots.

This ethologically inspired functional perspective also shows that there is actually no need to ‘climb’ the uncanny valley.

### Dogs are Showing the Way

The viability of this approach is strongly supported by an analogous situation existing between humans and dogs for more then 18,800–32,100 years (e.g., Thalmann et al., 2013). The domestication of the dogs (from a wolf-like ancestor) resulted in several important morphological and behavior changes in dogs that enhanced the possibility of dog-human social interaction (Hare and Tomasello, 2005; Miklósi, 2014). Further steps in dog evolution led to dog breeds which occupy specific behavioral niches with regard to their specific function in collaborative interactions with humans (Miklósi, 2014). The large number of dogs sharing our life as companions, or working individuals (e.g., rescue dogs, dogs leading bind persons) shows the success of this evolutionary change. Thus with regard to the above points both dogs and humans retained their independent capacity to evolve, dogs have changed and can change if novel niches for interaction with humans emerge (e.g., Gácsi et al., 2013) and there is only limited competition between the two species.

Importantly, there are two critical features of the domestication process. Because of biological constrains (e.g., reproduction) dogs retained basic morphology and behavior of their ancestors but at the same time they acquired a level of social competence that allows them to be integrated into the human society (Miklósi and Topál, 2013). The history of dogs shows that humans are able to interact in very sophisticated ways with agents that are morphologically and behaviorally rather different, but show a specific human-like social competence. Dogs’ social competence manifests in several cognitive domains including attachment, gestural and auditory inter-specific communication, inter-specific cooperation, ability to learn by observation (Topál et al., 2009b). Importantly, these components are supported by rather different mental mechanisms in dogs, and may show some important limitations when compared to analogous human skills (Lakatos et al., 2009; Topál et al., 2009a; Fugazza and Miklósi, 2014). Nevertheless, the connection and synergism that exists among these components lead to complex social competence in dogs, which allows them to perform efficiently in our societies.

### Social Competence in Robots

Earlier we defined social competence as an individual’s ability to generate social skills that conform to the expectations of others and the social rules of the group (Miklósi and Topál, 2013). Such complex level of interaction emerges if the individual wants to participate, has the means to participate, and is regarded by others as being able to participate in the life of the group (see also Johnson, 2001). Thus the overarching goal for social robots is to gain some level of social competence that allows them to be integrated in the human group.

Several research teams in the field of social robotics have aimed to define the necessary and sufficient skills for such agents. Such approaches are problematic because they regard the components of human social competence as a starting point. For example, Fong et al. (2003) provide a long list of quite specific human skills that social robots should possess. Apart from the fact that at the moment there is no robust technical solution available for most of these social skills, the human model is less appropriate here because the biological foundations of the social interaction are obscured by the complexity of our social and cultural behaviors.

### Bottom up Approach for Social Robotics

We suggest an alternative approach for the development of social robots using principles of dog-human interaction. First, the human-robot relationship should be represented as an inter-specific relationship rather than in an intra-specific one. As indicated above, such relationship is not unique among agents, and would most likely manifest some form of symbiosis in which humans experience positive fitness consequences (mutualism). Such functional approach to social robotics may also be helpful because it stresses that robots are constructed for a social process and not for a social state. Just like in the case of human-dog relationship, a social robot does not automatically become a social partner (e.g., companion) but it achieves this state of social affairs if it engages in the appropriate kind of social interactions with its partner (see Fujita, 2007; Miklósi and Gácsi, 2012). Any type of partnership is not an *a priori* attribute of the robot but actually an outcome of relevant social interactions between the agents. Accordingly, the social skills of the robot and the time devoted to the social interactions (by both parties) determine whether some type of partnership emerges or not.

We envisage that social robots should be able to show some basic social skills that are present in dogs. These may include, for example, attachment to humans (Topál et al., 2005), simple ways of communicative interaction (Miklósi et al., 2000; Gaunet and El Massioui, 2014), responsiveness to learning and training (Topál et al., 2009a) and being useful in some specific way (Naderi et al., 2001; Ostojic and Clayton, 2014). These commonalities between human and robot social competence are enough to form a basis for social interaction if there is time to gain experience mutually.

Importantly, there is no need to socialize humans to such social robots in any specific way or at any specific age and there is also no danger that humans develop unnatural preferences toward them.

## Promises of Ethorobots

Social robotics aims to deliver various robots that serve human needs in modern societies but society may not accept many present day social robots because of their limited abilities which contradict their human-like appearance. We argue that ethorobotics offers a new approach by suggesting that social robots should be regarded as separate species that are highly adapted to their niche, and their similarity to humans both in terms of physical appearance and behavior in itself (without specific function) is irrelevant. This also includes that social robots can and should have human like features if this is required and optimal for their functions (e.g., simple verbal feedback, or human hand).

Simple insights from ethology can lead to a new generation of social robots. Ethorobots’ basic social competence should ensure that humans eventually develop a social relation to them, which is sufficient for advantageous cooperation. We expect that these new ethorobots provide several advantages for the human society while avoiding possible dangers which may emerge if the present trend of technical development continues.

From the robots’ perspective:

(1) Ethorobots are more efficient in their own niche because they are not constrained by expected similarity to humans.
(2) Considering the state of art in robotics, ethorobots are more acceptable social partners than imperfect androids.
(3) Ethorobots do not pose the problem of having a gender because they could be still regarded as part of the category of animals, where the actual gender is of secondary importance from the human point of view.

From the humans’ perspective:

(1) Humans do not need to compete with ethorobots, instead, these robots would need to compete with each other (which of them is better at fulfilling a specific function).
(2) Humans can maintain control over ethorobots by controlling the nature of interaction, and whether they maintain or close down the actual niche for the robot.
(3) Humans have the necessary mental skills to learn to adjust their social behavior to robots with different embodiment and behavior if they show basic levels of social competence.

The validity and relevance of our claims and arguments can be tested by carrying out experiments that address the following questions. What is the minimally functioning social competence in ethorobots? Does it depend on embodiment and/or function? Would ethorobots be easier to accept by humans than humanoids, androids and any other type of human-like robots? What decides if embodiment and social behavior contradict or complement each other? Would humans develop different type of social relationships with ethorobots depending on their social competence? Under what condition would humans perceive an ethorobot as a living being? Experiments get started (e.g., Faragó et al., 2014; Lakatos et al., 2014; Takahashi et al., 2015; Gácsi et al., 2016; Paetzel et al., 2016; Tschöpe et al., 2017) but there is a long way to go.

## Conclusion

Robotics has reached a stage when there is a demand for robots that can be considered as partners of humans. But without a clear theory built on biological (ecological and technological) knowledge, social robotics may fall in serious traps, will not be able to fulfill the societies’ demand, and waste much money. We suggest robots that are developed on the basis of ethological concept: they (1) do not destroy natural human relationships, (2) do not get into a competitive situation with humans, (3) are able to develop a social partnership with humans, which matches the level of cooperation needed, and (4) are more acceptable for integration into our communities.

## Author Contributions

ÁM and MG: conception of the paper, drafting the work. PK and VM: conception of the paper, revising the draft. All: final approval of the version to be published, agreement to be accountable for all aspects of the work.

## Conflict of Interest Statement

The authors declare that the research was conducted in the absence of any commercial or financial relationships that could be construed as a potential conflict of interest.

## Footnotes

1 http://ieet.org/index.php/IEET/more/2083
